# Supplementary material for: New Drug Targets to Prevent Death Due to Stroke: A Review Based on Results of Protein-Protein Interaction Network, Enrichment, and Annotation Analyses
Source: Int J Mol Sci. 2021 Nov 9;22(22):12108. doi: 10.3390/ijms222212108 (PMC8619767; doi:10.3390/ijms222212108)
Supplement: Supplementary file 1 [file ijms-22-12108-s001.zip › ijms-1395203-supplementary.pdf]

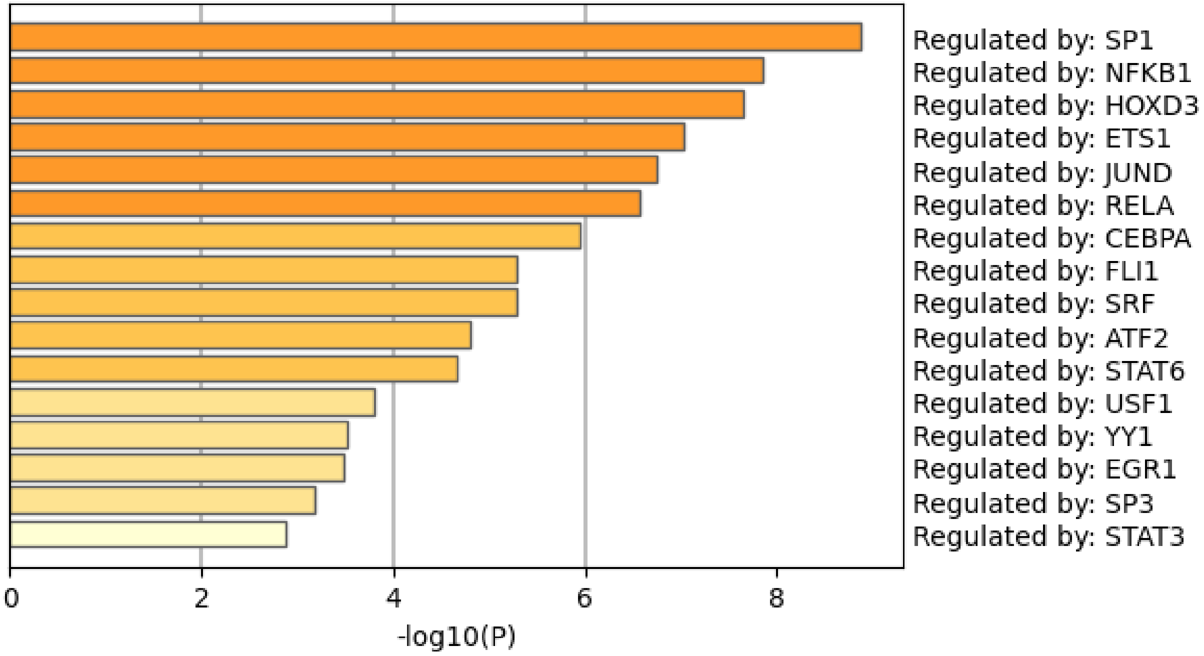
Regulated by: SP1
Regulated by: NFKB1
Regulated by: HOXD3
Regulated by: ETS1
Regulated by: JUND
Regulated by: RELA
Regulated by: CEBPA
Regulated by: FLI1
Regulated by: SRF
Regulated by: ATF2
Regulated by: STAT6
Regulated by: USF1
Regulated by: YY1
Regulated by: EGR1
Regulated by: SP3
Regulated by: STAT3
0
2
4
6
8
-log10(P)

ESF1 Figure S1. Heatmap of the GO_TTRUST transcriptional factors that were enriched in the immune cluster 1 (using Metascape) showing that regulated by SP1 and NFKB1 were the top transcription factor targets.

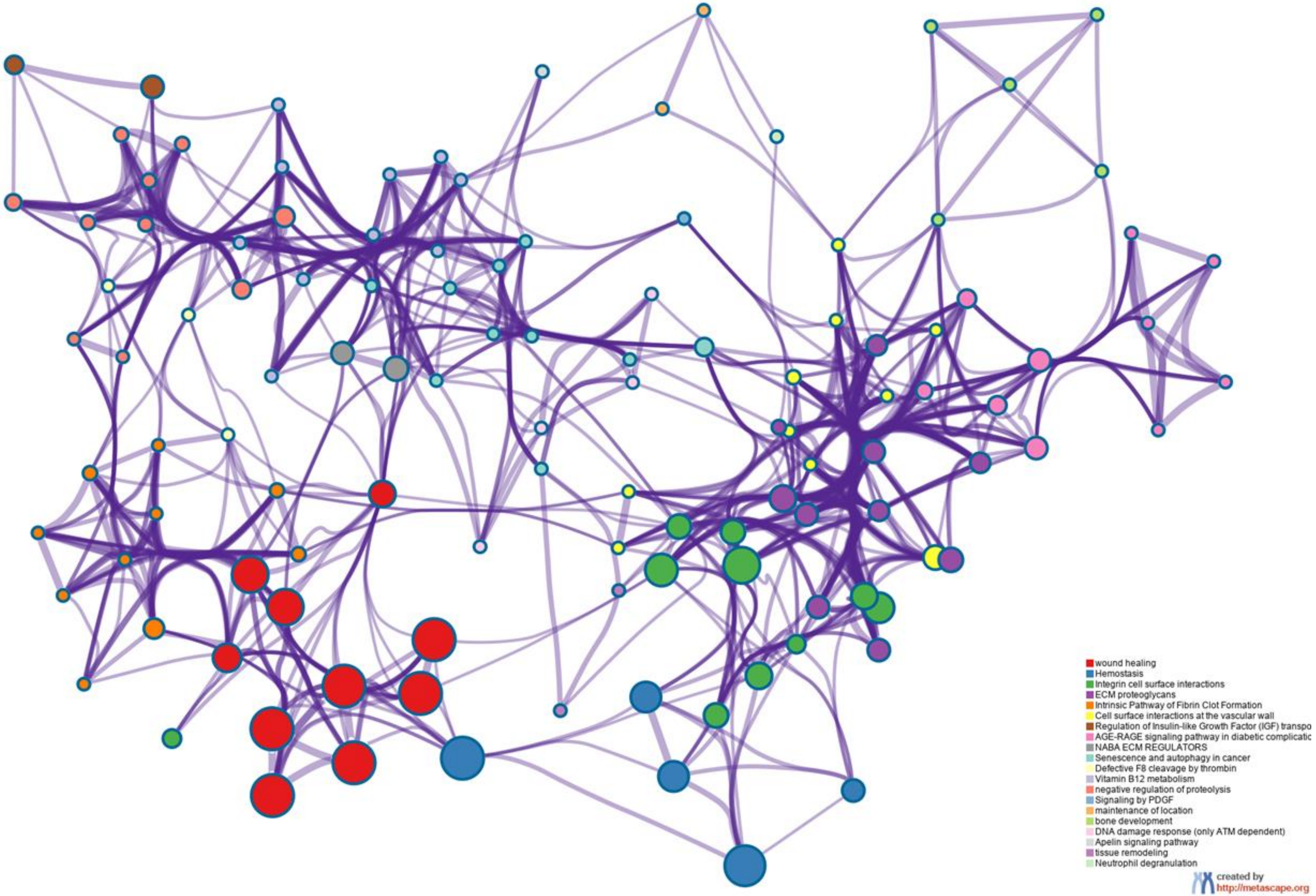
wound healing
Hemostasis
Integrin cell surface interactions
ECM proteoglycans
Intrinsic Pathway of Fibrin Clot Formation
Cell surface interactions at the vascular wall
Regulation of Insulin-like Growth Factor (IGF) transpo
AGE-RAGE signaling pathway in diabetic complicatic
NABA ECM REGULATORS
Senescence and autophagy in cancer
Defective F8 cleavage by thrombin
Vitamin B12 metabolism
negative regulation of proteolysis
Signaling by PDGF
maintenance of location
bone development
DNA damage response (only ATM dependent)
Apelin signaling pathway
tissue remodeling
Neutrophil degranulation
created by
http://metascape.org

ESF1 Figure S2. Enriched ontology term clusters in the hemostasis subnetwork of death due to ischemic stroke. Terms are represented by a circle node, with the colors representing cluster identity and their size reflecting the number of input genes. One term is used to describe the clusters (colored labels). The thickness of the (bundled) edges linking the terms reflects the similarity score (threshold is > 0,3). The network is constructed and visualized using MetaScape and Cytoscape (v3.1.2).

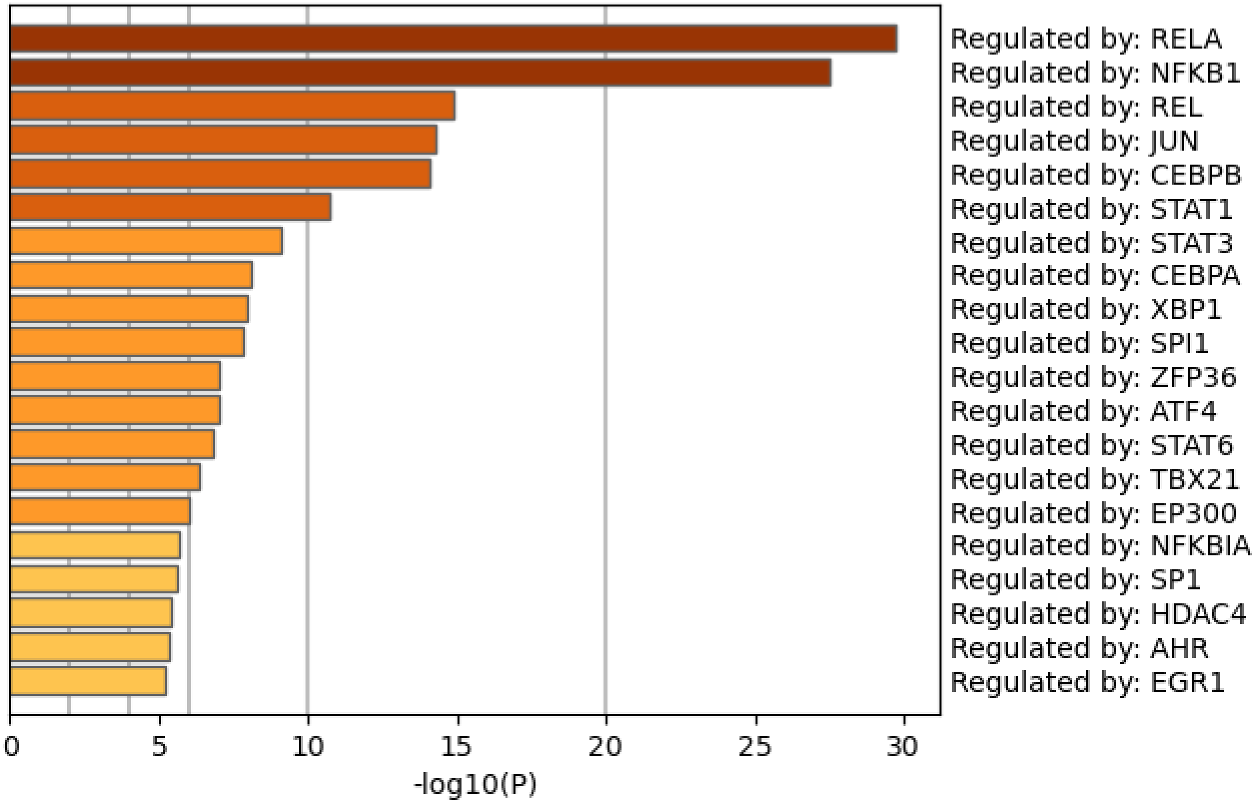
Regulated by: RELA
Regulated by: NFKB1
Regulated by: REL
Regulated by: JUN
Regulated by: CEBPB
Regulated by: STAT1
Regulated by: STAT3
Regulated by: CEBPA
Regulated by: XBP1
Regulated by: SPI1
Regulated by: ZFP36
Regulated by: ATF4
Regulated by: STAT6
Regulated by: TBX21
Regulated by: EP300
Regulated by: NFKBIA
Regulated by: SP1
Regulated by: HDAC4
Regulated by: AHR
Regulated by: EGR1
0
5
10
15
20
25
30
-log10(P)

ESF1 Figure S3. Bar graph with the top-16 transcriptional factors (TTRUST) which were enriched in the hemostasis subnetwork indicating that regulated by RELA and NFKB1 were top transcription factor targets.

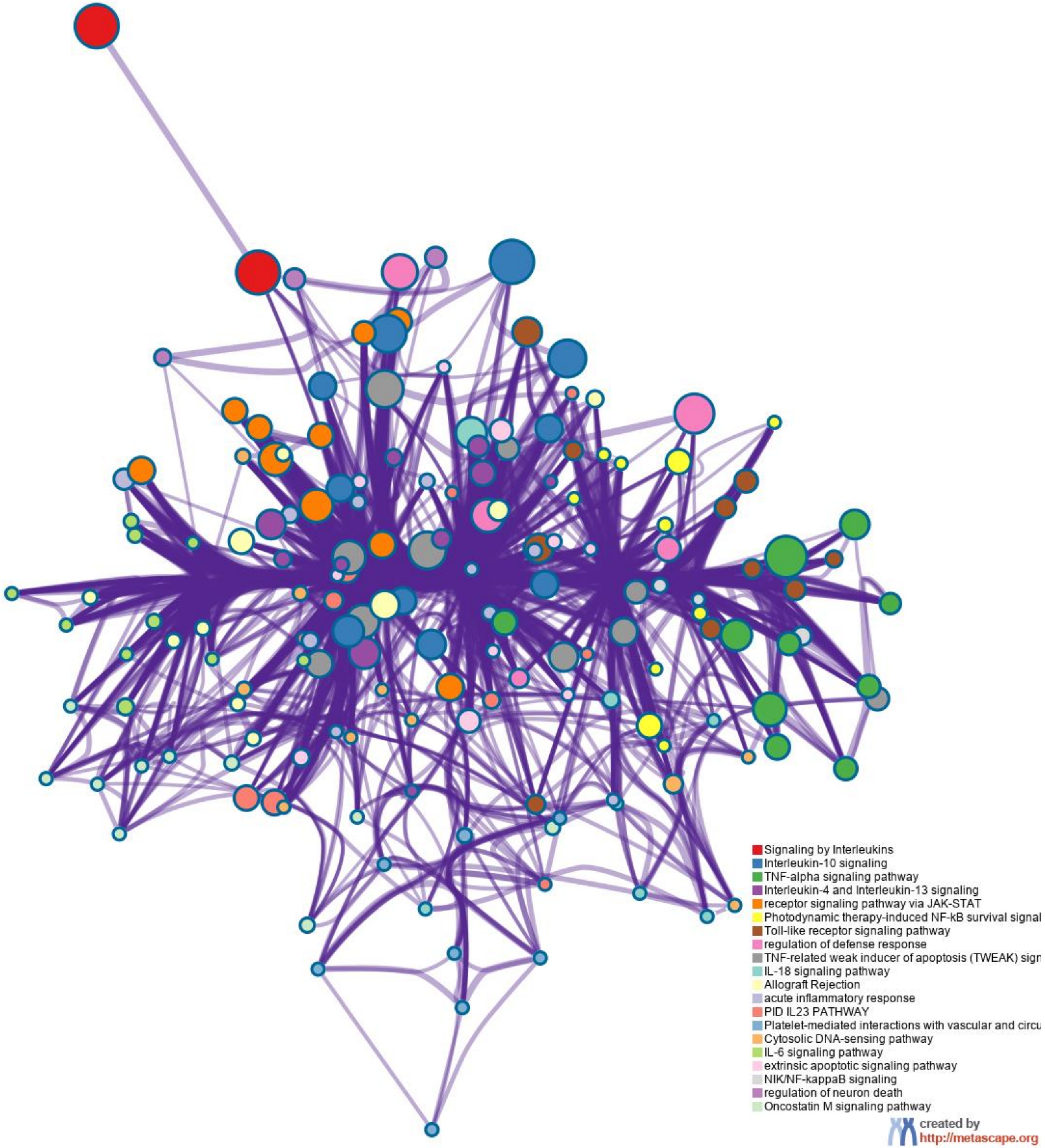
Signaling by Interleukins
Interleukin-10 signaling
TNF-alpha signaling pathway
Interleukin-4 and Interleukin-13 signaling
receptor signaling pathway via JAK-STAT
Photodynamic therapy-induced NF-kB survival signal
Toll-like receptor signaling pathway
regulation of defense response
TNF-related weak inducer of apoptosis (TWEAK) sigr
IL-18 signaling pathway
Allograft Rejection
acute inflammatory response
PID IL23 PATHWAY
Platelet-mediated interactions with vascular and circu
Cytosolic DNA-sensing pathway
IL-6 signaling pathway
extrinsic apoptotic signaling pathway
NIK/NF-kappaB signaling
regulation of neuron death
Oncostatin M signaling pathway
created by
http://metascape.org

ESF1 Figure S4. Enriched ontology term clusters in the immune subnetwork of death due to ischemic stroke. Terms are represented by a circle node, with the colors representing cluster identity and their size reflecting the number of input genes. One term is used to describe the clusters (colored labels). The thickness of the (bundled) edges linking the terms reflects the similarity score (threshold is > 0,3). The network is constructed and visualized using MetaScape and Cytoscape (v3.1.2).

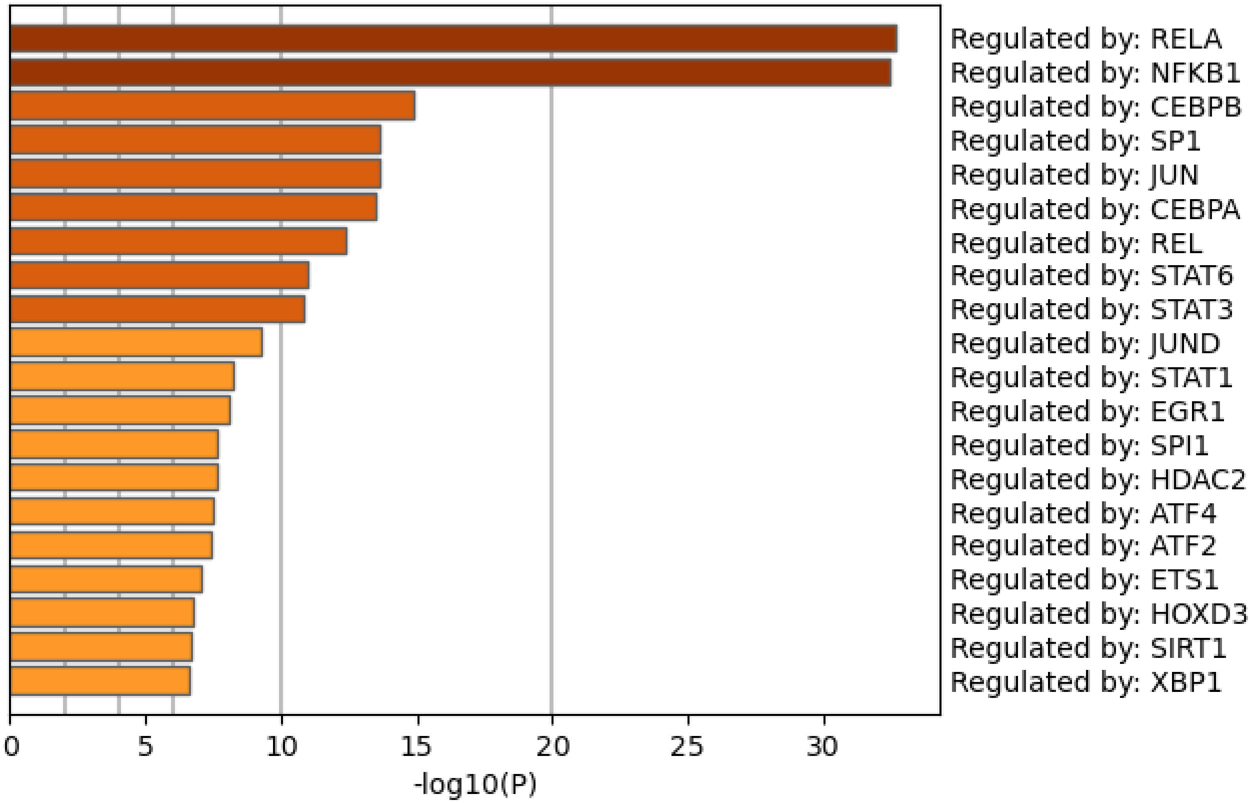
Regulated by: RELA
Regulated by: NFKB1
Regulated by: CEBPB
Regulated by: SP1
Regulated by: JUN
Regulated by: CEBPA
Regulated by: REL
Regulated by: STAT6
Regulated by: STAT3
Regulated by: JUND
Regulated by: STAT1
Regulated by: EGR1
Regulated by: SPI1
Regulated by: HDAC2
Regulated by: ATF4
Regulated by: ATF2
Regulated by: ETS1
Regulated by: HOXD3
Regulated by: SIRT1
Regulated by: XBP1
0
5
10
15
20
25
30
-log10(P)

ESF1 Figure S5. Heatmap with the top TTRUST factors were enriched in all differentially expressed proteins and genes indicating that regulated by RELA and NFKB1 were the top transcription factor targets.

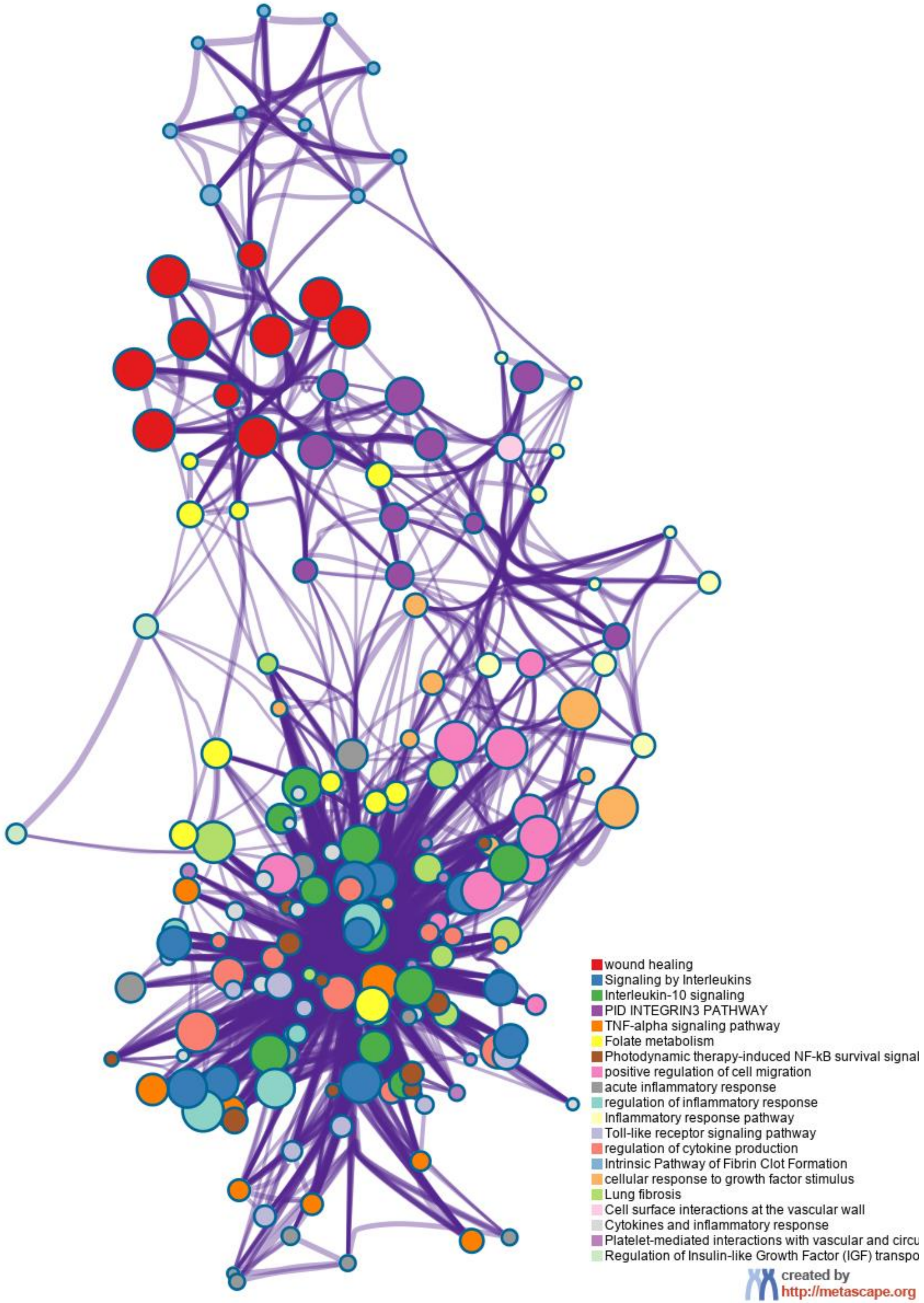
wound healing
Signaling by Interleukins
Interleukin-10 signaling
PID INTEGRIN3 PATHWAY
TNF-alpha signaling pathway
Folate metabolism
Photodynamic therapy-induced NF-kB survival signal
positive regulation of cell migration
acute inflammatory response
regulation of inflammatory response
Inflammatory response pathway
Toll-like receptor signaling pathway
regulation of cytokine production
Intrinsic Pathway of Fibrin Clot Formation
cellular response to growth factor stimulus
Lung fibrosis
Cell surface interactions at the vascular wall
Cytokines and inflammatory response
Platelet-mediated interactions with vascular and circu
Regulation of Insulin-like Growth Factor (IGF) transpo
created by
http://metascape.org

ESF1 Figure S6. Enriched ontology term clusters in the death due to ischemic stroke network based on all differentially expressed proteins and genes. Terms are represented by a circle node, with the colors representing cluster identity and their size reflecting the number of input genes. One term is used to describe the clusters (colored labels). The thickness of the (bundled) edges linking the terms reflects the similarity score (threshold is > 0,3). The network is constructed and visualized using MetaScape and Cytoscape (v3.1.2).

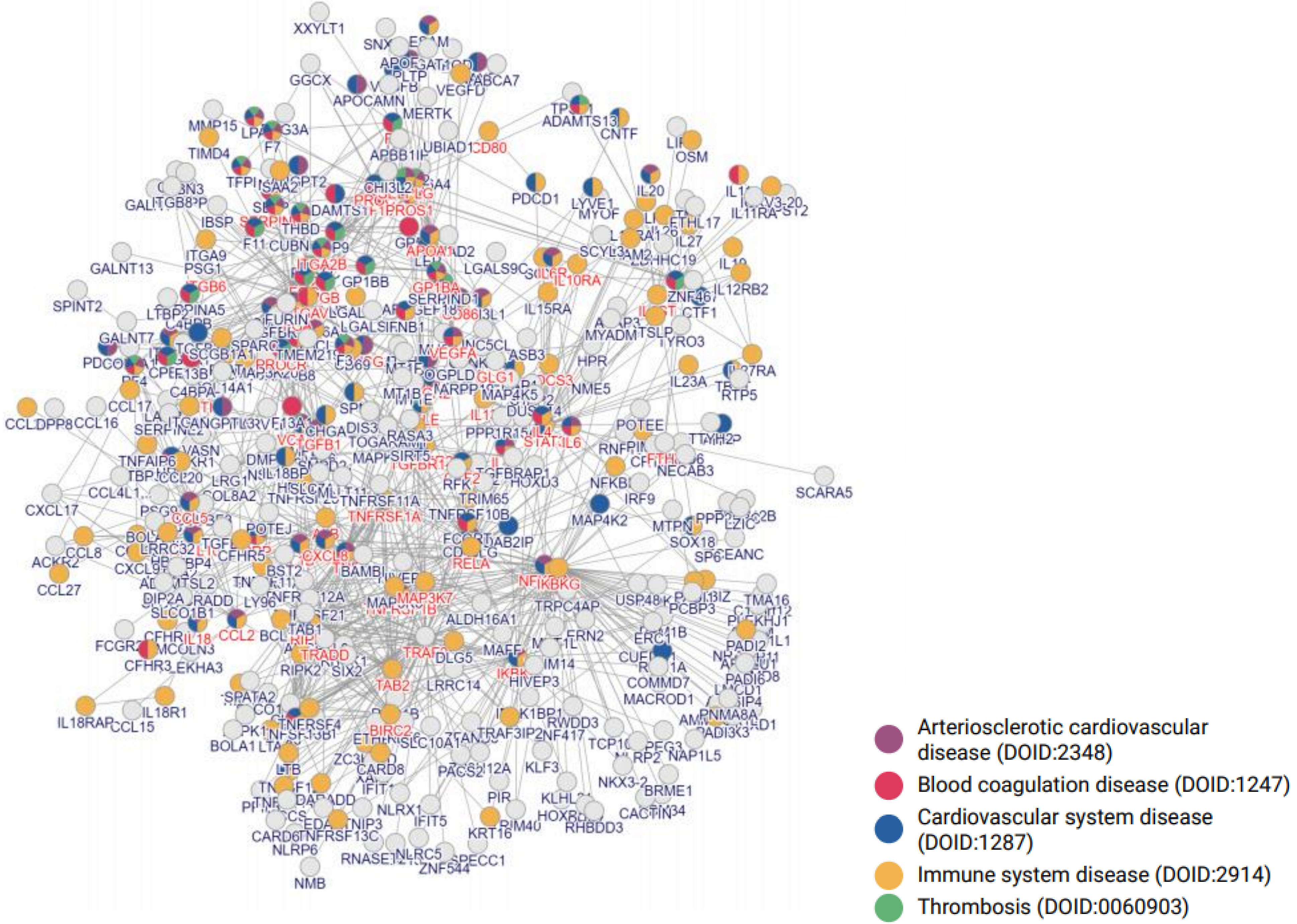
Arteriosclerotic cardiovascular disease (DOID:2348)
Blood coagulation disease (DOID:1247)
Cardiovascular system disease (DOID:1287)
Immune system disease (DOID:2914)
Thrombosis (DOID:0060903)
XXYLT1
GGCX
APOCAMN
MERTK
VEGFD
ABCA7
ADAMTS13
CNTF
OSM
CD80
UBIAD1
APBB1IP
MMP15
TIMD4
PDCD1
IL20
IL11RA
GALNT13
SPINT2
GALNT7
ITGA9
PSG1
IBSP
THBD
CUBN
ITGA2B
GP1BB
GP1BA
LGALS9C
IL10RA
IL15RA
IL12RB2
IL27
SCYL3
TSLP
TYRO3
MYADM
ASB3
HPR
NME5
IL23A
RTP5
IL27RA
VEGFA
PROCR
SCGB1A1
LTBP2
CCL17
CCL16
DPP8
CHGA
TGFB1
DIS3
RASA3
SIRT5
TOGARAM1
STAT3
IL6
POTEE
FTH1
NECAB3
SCARA5
TNFAIP6
CCL20
LRG1
COL8A2
TNFRSF11A
TNFRSF1A
TNFRSF10B
TGFBRAP1
HOXD3
TRIM65
NFKBIA
IRF9
MAP4K2
MTPN
SOX18
SPOCK1
CXCL17
CCL4L1
CCL5
CCL8
ACKR2
CCL27
POTEJ
CFHR5
BST2
BAMBI
CXCL8
RELA
DAB2IP
NFKB1
IKBKG
USP48
PCBP3
TMA16
PADI2
DIP2A
RADD
LY96
SLCO1B1
MAP3K7
ALDH16A1
TRPC4AP
FRN2
ERC1
FCGR2A
CFHR3
MCOLN3
PLEKHA3
IL18
CCL2
TRADD
TAB2
TAB1
DLG5
MAFF
LRRC14
HIVEP3
COMMD7
MACROD1
IL18RAP
CCL15
IL18R1
SPATA2
BOLA1
LTA
TNFRSF4
BIRC2
SLC10A1
TRAF3IP2
ZNF417
RWDD3
TCP10
NAP1L5
LTB
CARD8
ZC3H12A
PACS2
KLF3
NKX3-2
BRME1
CACTIN
RHBDD3
CARD6
TNFRSF13C
NLRP6
NLRX1
IFIT5
TNIP3
KRT16
PIR
RNASE1
NLRC5
ZNF544
SPECC1
NMB
LRRC32
ITGB6
SERPINA5
CFHR1
CCL27

ESF1 Figure S7. An extended network constructed with inBio Discover showing the top Disease Ontology (DOID) annotations.

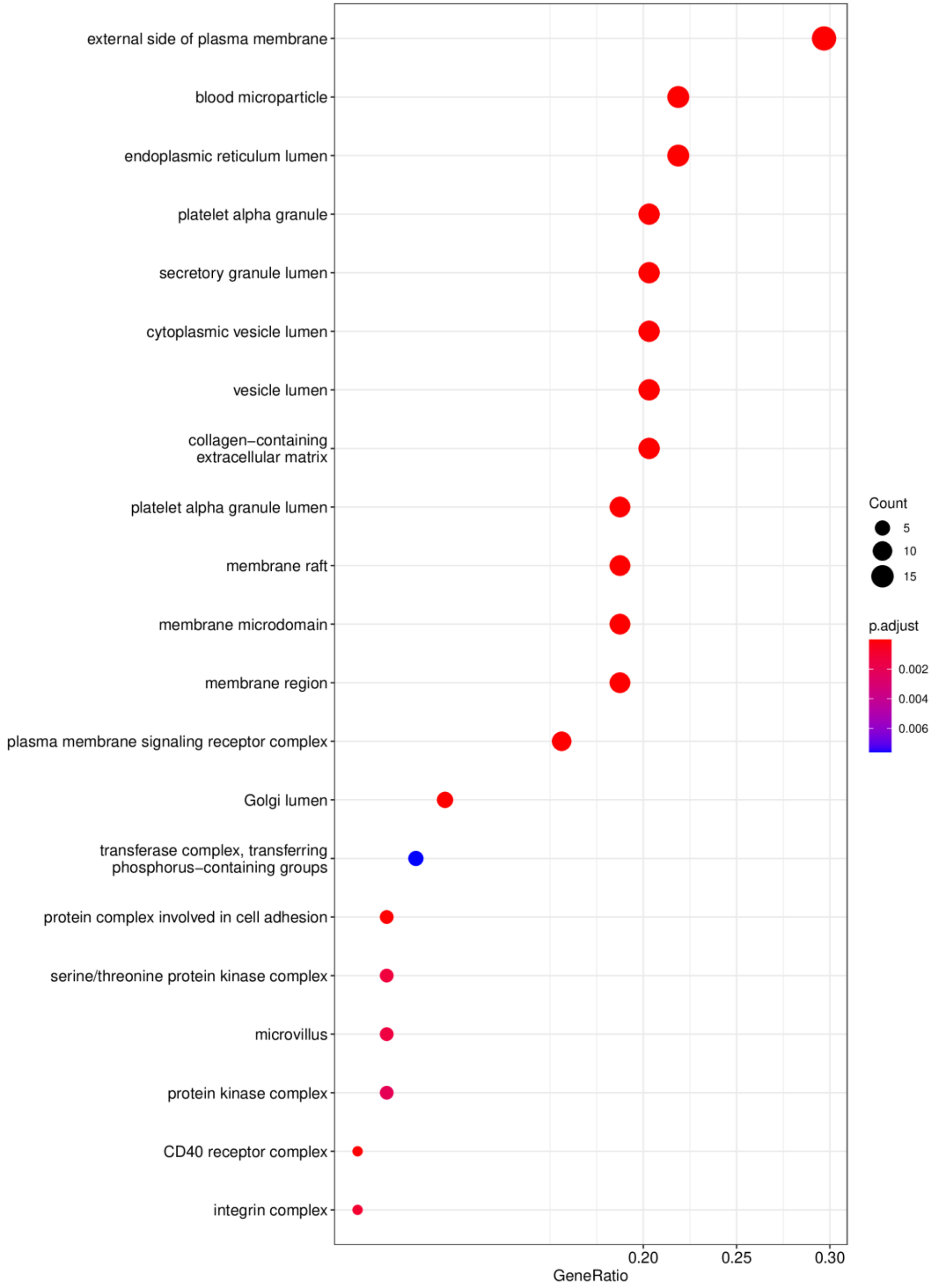

external side of plasma membrane
blood microparticle
endoplasmic reticulum lumen
platelet alpha granule
secretory granule lumen
cytoplasmic vesicle lumen
vesicle lumen
collagen−containing extracellular matrix
platelet alpha granule lumen
membrane raft
membrane microdomain
membrane region
plasma membrane signaling receptor complex
Golgi lumen
transferase complex, transferring phosphorus−containing groups
protein complex involved in cell adhesion
serine/threonine protein kinase complex
microvillus
protein kinase complex
CD40 receptor complex
integrin complex
0.20
0.25
0.30
GeneRatio
Count
5
10
15
p.adjust
0.002
0.004
0.006

ESF1 Figure S8. GO functional enrichment analysis of the cellular components of all differently expressed proteins and genes using R package ClusterProfiler. The x-axis shows the gene ratio and the y-axis the annotated terms. The size of the dots is proportional to the gene number; p-values of all GO terms are colored as indicated in the figure.

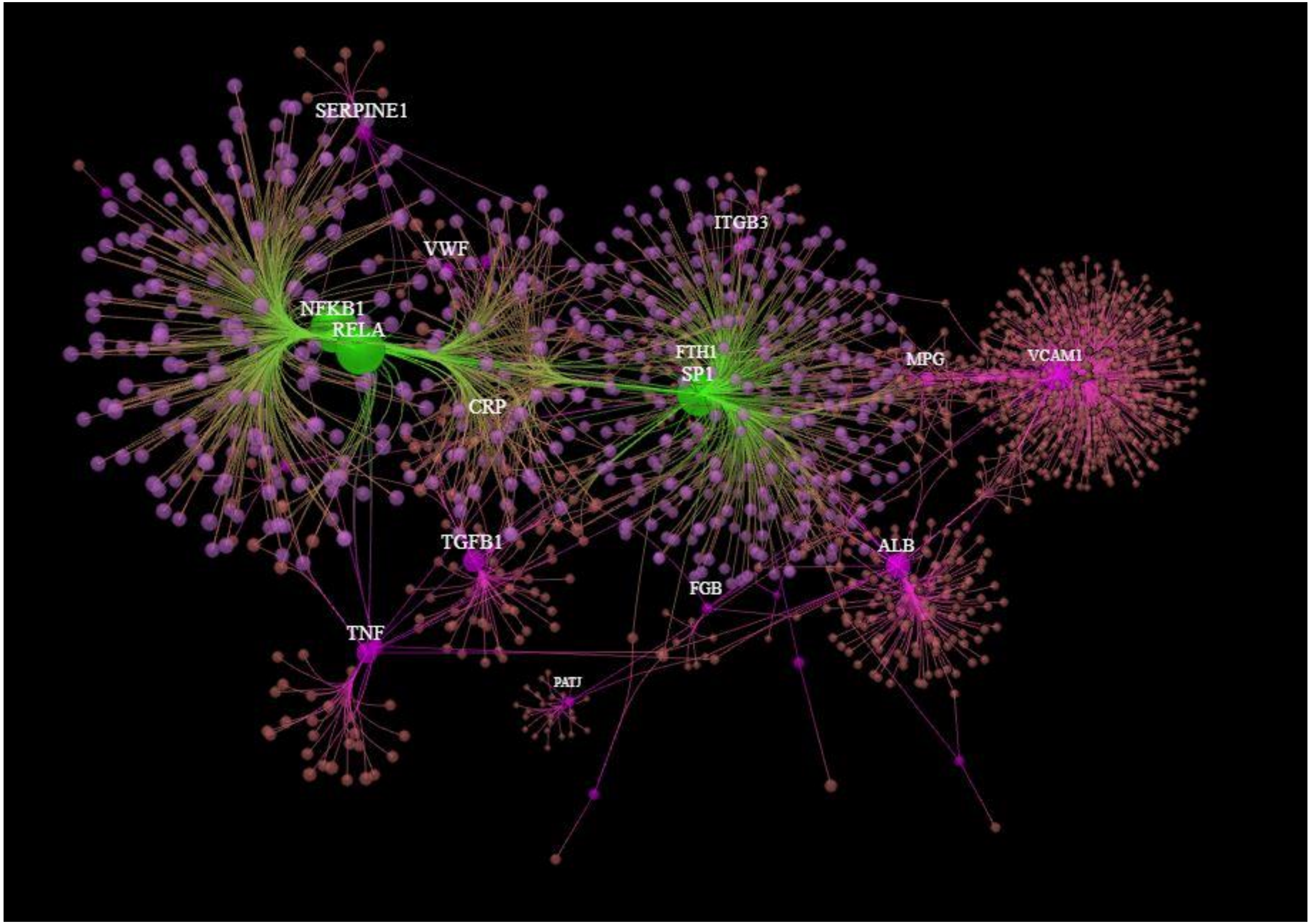
SERPINE1
ITGB3
VWF
NFKB1
RELA
FTH1
SP1
MPG
VCAM1
CRP
TGFB1
ALB
FGB
TNF
PATJ

ESF1 Figure S9. The targeted integration of three regulating actors, namely NFKB, RELA and SP1 (shown in green colors) in the network of death due to ischemic stroke. This MultiOmics network (using InAct, mirNET, and TTRUST) is built using differentially expressed proteins, genes, and miRNA using OmicsNet.

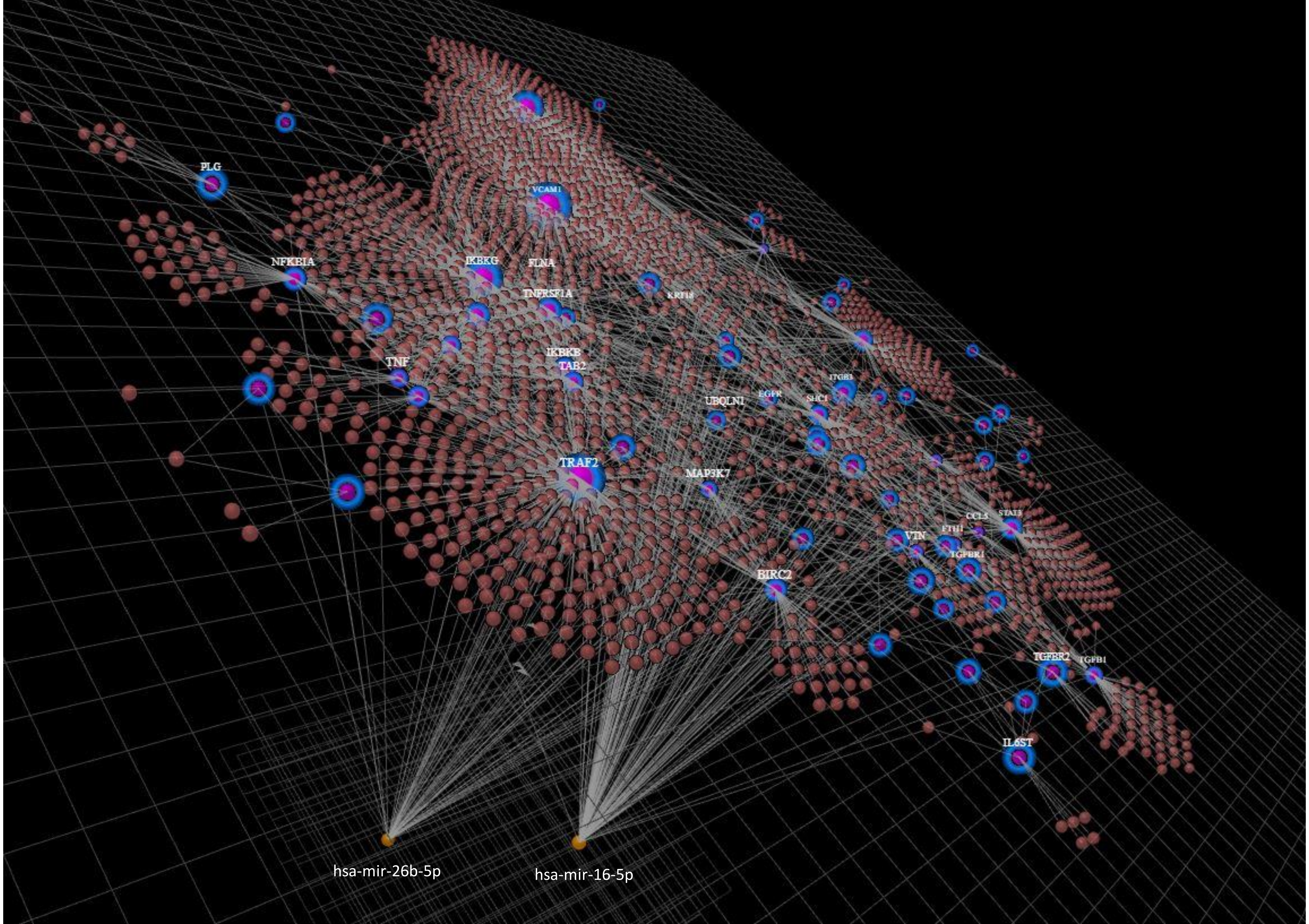
PLG
VCAM1
NFKBIA
IKBKG
FLNA
TNFRSF1A
KRT18
IKBKB
TAB2
TNF
UBQLN1
EGFR
SHC1
ITGB3
TRAF2
MAP3K7
STAT3
CCL5
FTH1
VTN
TGFBR1
BIRC2
TGFBR2
TGFB1
IL6ST
hsa-mir-26b-5p
hsa-mir-16-5p

ESF1 Figure S10. The targeted integration of two miRNA regulating actors, namely hsa-mir-16-5p and hsa-mir-26b-5p in the network of death due to ischemic stroke. This MultiOmics network (using InAct, mirNET, and TTRUST) is built using differentially expressed proteins, genes, and miRNA using OmicsNet.
